# Epstein-Barr Virus Limits the Accumulation of IPO7, an Essential Gene Product

**DOI:** 10.3389/fmicb.2021.643327

**Published:** 2021-02-16

**Authors:** Ya-Chun Yang, Bill Sugden

**Affiliations:** McArdle Laboratory for Cancer Research, University of Wisconsin-Madison, Madison, WI, United States

**Keywords:** epstein-barr virus, miRNAs, IPO7, EGR1, essential gene

## Abstract

Epstein-Barr virus (EBV) encodes more than 40 miRNAs that target cellular mRNAs to aid its infection, replication, and maintenance in individual cells and in its human host. Importin-7 (IPO7), also termed Imp7 or RanBPM7, is a nucleocytoplasmic transport protein that has been frequently identified as a target for two of these viral miRNAs. How the viral life cycle might benefit from regulating IPO7 has been unclear, though. We demonstrate with CRISPR-Cas9 mutagenesis that IPO7 is essential in at least three cells lines and that increasing its levels of expression inhibits growth of infected cells. EBV thus regulates the level of IPO7 to limit its accumulation consistent with its being required for survival of its host cell.

## Introduction

Epstein-Barr virus (EBV) is a human herpesvirus that is associated with several human cancers, including Burkitt’s lymphoma (BL), Hodgkin’s disease, post-transplant lymphoproliferative disease, and both gastric and nasopharyngeal carcinoma (NPC; Raab-Traub, 2002, 2015; Hammerschmidt and Sugden, 2004; Brady et al., 2007; Sugden, 2014; Tse and Kwong, 2015). Rather than only infecting replicating cells, EBV can infect quiescent B cells and drive them to proliferate. While EBV genomes are maintained as plasmids in these proliferating cells, defects in its DNA synthesis lead to its being lost from a population of proliferating cells unless it provides the cells that retain it sufficient selective advantages to outgrow those that lose it (Kennedy and Sugden, 2003; Nanbo et al., 2007; Vereide and Sugden, 2009). Multiple studies have shown that EBV’s miRNAs provide selective advantages early in infection of B-cells, in B-cell tumors, and in the switch from latency to the lytic phase of EBV’s life cycle (Feederle et al., 2011; Vereide et al., 2014; Albanese et al., 2016; Bernhardt et al., 2016; Tagawa et al., 2016; Chen et al., 2019). EBV encodes ~40 miRNAs, four of which are expressed from the BHRF locus, the remainder are expressed from the BART locus. These processed, mature miRNAs have been shown to regulate both cellular and viral functions (Cai et al., 2006; Barth et al., 2011; Kuzembayeva et al., 2014; Lyu et al., 2018). The BART cluster is expressed in all EBV-positive proliferating cells examined (Pfeffer et al., 2004; Skalsky et al., 2012). The BART miRNAs foster EBV’s life cycle by inhibiting apoptosis and immune responses to infected B-cells (Marquitz et al., 2011; Vereide et al., 2014; Lin et al., 2015; Albanese et al., 2016; Tagawa et al., 2016; Murer et al., 2019). The BART miRNAs also likely promote epithelial cell survival by targeting several proapoptotic cellular genes and could thereby contribute to the EBV-mediated epithelial carcinogenesis (Marquitz et al., 2012, 2014; Cai et al., 2015; Kanda et al., 2015; Kang et al., 2015; Shinozaki-Ushiku et al., 2015; Zhang et al., 2018; Wang et al., 2019).

Multiple studies have identified the transcripts of IPO7 as common targets for EBV’s miRNAs including BART3 and BART16 (Riley et al., 2012; Skalsky et al., 2012; Vereide et al., 2014). These identifications have been supported by conditional assays and genetic analyses. For example, we have evicted EBV from Burkitt Lymphoma cells and found that their levels of the IPO7 protein increased. Re-expression of the viral miRNAs in these evicted cells decreased IPO7 to parental levels (Vereide et al., 2014). We have also infected primary B-cells with EBV and a derivative from which its miRNA genes were deleted and assayed the levels of IPO7 mRNAs. The absence of EBV’s miRNAs in newly infected B-cells led to a significant increase in the expression of IPO7 mRNA (Tagawa et al., 2016).

However, the selective advantages EBV gains by inhibiting IPO7 are unclear. IPO7, also called Imp7 or RanBPM7, is a nucleocytoplasmic transport protein that binds cargos in the cytoplasm, transports them through nuclear pore complexes (NPCs), and releases them in the nucleus upon binding to RanGTP (Görlich et al., 1997). IPO7 is an import factor for several proteins, including ribosomal proteins, histone H1, glucocorticoid receptor, Early growth response protein 1 (EGR1), HIV-1 integrase protein, NUP93, and SMAD4 (Jäkel and Görlich, 1998; Fassati et al., 2003; Freedman and Yamamoto, 2004; Ao et al., 2007; Chen et al., 2011; Kang et al., 2013; Braun et al., 2016). Inhibiting IPO7 decreases the nuclear import of some ribosomal proteins leading to the activation of cell death in some cells (Golomb et al., 2012). This finding might mean that EBV’s targeting IPO7 with its miRNAs inhibits survival of its transformed cells, a notion at odds with EBV’s known roles in transformation. We therefore sought to understand what advantages EBV gains by targeting IPO7. We found unexpectedly that IPO7 is an essential gene in an uninfected cell line and in both EBV-infected lymphoid and epithelial tumor cells. In addition, decreasing the expression of IPO7 in these cells did not affect their proliferation, while increasing it in EBV-positive gastric carcinoma cells inhibited their growth. These changes in the growth of the cells were paralleled by changes in IPO7’s activity as shown, for example, by the levels of EGR1 imported into the nucleus. EBV thus limits the level of IPO7 through its miRNAs to optimize the growth of transformed cells.

## Materials and Methods

### Cell Lines

Epstein-Barr virus-positive SNU-719 cells, a gastric carcinoma-derived cell line (Oh et al., 2004), and 721 cells, a B-cell line transformed by EBV *in vitro* (Kavathas et al., 1980), were cultured in RPMI 1640 (Invitrogen) supplemented with L-glutamine, 10% fetal bovine serum (FBS), and antibiotics (200 U/ml penicillin and 200 μg/ml streptomycin). 293T cells were cultured in DMEM (Invitrogen) supplemented with L-glutamine, 10% FBS, and antibiotics (200 U/ml penicillin and 200 μg/ml streptomycin).

### Plasmids

Plasmid 3051 was a retroviral vector that was constructed as described previously (Kennedy and Sugden, 2003). 3051-IPO7 was generated by cloning an IPO7 cDNA sequence into the NotI/BamHI sites of 3051. Plasmids pJWB1157 and pCEP-CRISPR that were used for generating CRISPR-Cas9-containing plasmids were kindly provided by Dr. Eric Johannsen (Department of Medicine, School of Medicine and Public Health, University of Wisconsin-Madison, Madison, WI, United States).

### CRISPR-Cas9-Mediated Genome Editing

The targeting regions in IPO7 genomic DNA were predicted with the CRISPR Design Tool from the Zhang Lab (Hsu et al., 2013). The 20 n.t. spacer sequence followed by 3 n.t. PAM sequence (NGG) was inserted into the BbsI site of vector pJWB1157, which contains the Cas9 coding region and a U6-promoter regulated guide RNA. The cassette containing Cas9 and targeting sequence along with a guide RNA was digested with PciI/NotI and inserted into pCEP-CRISPR, which encodes EBNA1, OriP, and resistance to hygromycin. The plasmid pCEP-target-CRISPR was transfected into 293T, SNU719, or 721 cells, the cells selected with Hygromycin (200–500 μg/ml), clones isolated, and characterized *via* DNA sequencing.

### Cell Transfection

293T and SNU-719 cells were transfected with different plasmid DNAs using Lipofectamine 2000 (Invitrogen) following the manufacturer’s instructions. About 721 cells were transfected by electroporation. The transfected cells were cultured for 2 days before any following analysis.

### Cell Cloning

About 30, 100, 300, and 1,000 SNU-719 cells containing 3015 or 3015-IPO7 were seeded into 10 cm plates. After 1 month, the clones were counted, and the cloning frequency was calculated by dividing the number of surviving clones by the number of cells that had been seeded.

### Western Blotting Analysis

About 5 × 10^6 cells were collected and lysed in RIPA buffer (50 mM Tris-HCl, pH 7.8, 50 mM NaCl, 5 mM EDTA, 0.5% Triton X-100, 0.5% NP-40) and Western blotting analysis was performed as described previously (Pratt et al., 2012). The blots were detected with mouse monoclonal anti-IPO7 (Sigma) directed to the C-terminus at 1:1,000 dilution, or mouse monoclonal anti alpha-tubulin (Sigma) at 1:10,000 dilution, followed by a secondary anti-mouse antibody (Promega) at 1:5,000 dilution.

### Retroviral Transduction

The retroviruses were derived from a vesicular stomatitis virus G protein pseudotyped murine leukemia virus (Ory et al., 1996). Retrovirus was generated by transfecting 293T cells with 3 μg of a plasmid encoding the Gag-Pol element, 1 μg of a plasmid encoding the vesicular stomatitis virus G protein, 1 μg of plasmid encoding a derivative of nuclear factor-NFkB, and 10 μg of a plasmid carrying the retroviral backbone containing either IPO7 or empty vector control using linear PEI reagent per 10 cm plate. Twenty-four hours post-transfection, the culture medium was changed with fresh DMEM supplemented with 50 mM HEPES. The supernatant was collected after 2–4 days post-transfection and filtered through a 0.45-micron filter. The virus particles were pelleted by centrifuging at 38,000 rpm for 3 h at 4°C and resuspending the pellet in 1/10 the initial volume of medium.

### Flow Cytometry

About 1 × 10^7 cells were trypsinized and washed with phosphate buffered saline (PBS) containing 1 mM EDTA, 25 mM HEPES, and 5% FBS to avoid cell aggregation. The cell suspension was filtered immediately prior to cell sorting. Cells were sorted on a BD FACSAria in a Biosafety cabinet. To obtain cells that expressed various levels of the particular transgene, cells were sorted based on the fluorescence intensity of EGFP expressed from an IRES downstream of the transgene.

### Immunofluorescent Detection of EGR1 Nuclear Translocation

About 1 × 10^6 SNU-719 cells transfected with 3051 or 3051-IPO7 were collected and washed with PBS. The cells were fixed in 4% paraformaldehyde (PFA) for 10 min, washed, permeabilized with 0.05% triton X, and incubated for 20 min with 1% BSA. The cells were further incubated with rabbit anti-EGR1 monoclonal antibody (1:200; Cell Signaling) in PBS containing 1% BSA for 40 min. After washing with PBS, the cells were incubated for 20 min with 0.2 μg PE-conjugated donkey anti-rabbit IgG (Bio Legend) in 200 μl of PBS/1% BSA. The cells were then washed with PBS and resuspended in 50 μl of PBS/2% FBS with Draq5 (1:2,000; Bio Legend) for 5 min and run directly on the ImageStream Mark II (Amnis).

### ImageStream Data Acquisition and Analysis

The fluorescence image-based method for quantifying nuclear translocation was described previously (George et al., 2006). The degree of nuclear translocation can be measured by a similarity score that compares a cell’s nuclear fluorescence image to the pattern of fluorescence produced by the EGR1 signal. A high positive value of a similarity score would indicate that the signal from EGR1 and the corresponding nuclear image are similar. The similarity score is derived from Pearson’s correlation coefficient (PCC, *ρ*) that has been validated previously (George et al., 2006). Briefly, the formula of the PCC is denoted as:

$$\rho =\frac{\sum_{i}\left(x_{i}-X\right)\left(y_{i}-Y\right)}{\sqrt{\sum_{i}{\left(x_{i}-X\right)}^{2}{\left(y_{i}-Y\right)}^{2}}}$$

where x_i_, y_i_ are the pre-pixel intensity values of the two images at the same location, and X, Y are the corresponding mean intensity values. In this study, the data sources are the nuclear and EGR1 fluorescence images of the same cell. The similarity score transforms the PCC to a log scale to improve the discrimination, which was denoted as:

$$Similarity=\ln\left(\frac{1+\rho }{1-\rho }\right)$$

### Reverse Transcription and Real-Time PCR

SNU-719 cells transfected with 3051 or 3051-IPO7 were sorted, the total RNA was isolated using TRIzol (Ambion) according to the manufacturer’s instructions and treated with DNase (QIAGEN) to remove contaminating DNA (Pratt et al., 2012). Around 300 ng of total RNA was reverse transcribed using AMV reverse transcriptase (Invitrogen) following the manufacturer’s instructions. Real-time PCR was conducted as previously described (Pratt et al., 2009; Yang et al., 2017) and the relative mRNA quantity were determined by relative quantification using untreated SNU-719 mRNA to prepare standard curves, and then normalized to GAPDH. The following oligonucleotide sequences were used for real-time PCR (IDT): GAPDH (forward primer: TCAACGACCACTTTGTCAAGCT, reverse primer: CCATGAGGTCCACCACCCT, probe: TTCCTGGTATGACAACGAATTTGGCTACAGC), BMF (forward primer: TGTGCAGGAAGAGGAGGAT, reverse primer: CGAAAGCTTCAGTGCATTGC, probe: CATGTGCAGCAACACCAGCAGAAC), PTPRO (forward primer: CACCAGCCCTAAGATGTCAAC, reverse primer: AGCAAGCTACCAAGAGCAAA, probe: AATGAATGTTCCTGTCCGTCCCACG), BLZF1 (forward primer: AGCTGCTCTTGAGTTGGTT, reverse primer: AGCTTTCTGAACAGTTAGAACGTA, probe: TCATCTGCCATTACCCTGCTTGCA), CASP7 (forward primer: GTGGTCTTGATGGATCGCAT, reverse primer: AGATTCAGTGGATGCTAAGCC, probe: TCCTCGTTTGTACCGTCCCTCTTCA), MVP (forward primer: ATCCAGAACCTCCTCAAACAC, reverse primer: CCATCATCAGGCAGAACCAG, probe: TCTTCCCCTGTCACCCTCTCCT), IPO7 (forward primer: TGTATCCTCCATGCTTCTCAATC, reverse primer: ACTGACTCACGGTCTTAATGAAG, probe: CAGCCAGAGTTGCTATGTCCTGTAACT), and EGR1 (forward primer: CTTTTCCCTTTCTTTCCCCTTT, reverse primer: TGTCACCAACTCCTTCAGC, probe: TGTCATGTCCGAAAGCCCTGTGG). The probe of GAPDH was labeled with 5' FAMRA and 3' TAMRA, and the other probes were labeled with 5' FAMRA and 3' Iowa Black.

## Results

### Both Alleles of IPO7 Could Not Be Deleted From Human Cell Lines

**Figure 1:**
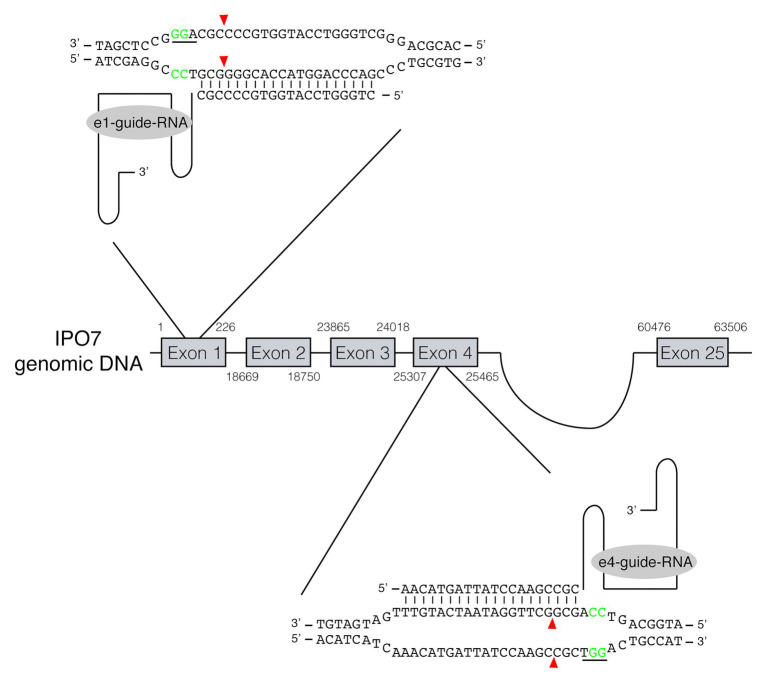
RNA-guided gene editing for the cellular protein IPO7. RNA-guided gene targeting in human cells involves guided RNAs expressed from U6 promoter that target the genomic sequence in the presence of a PAM at its 3'-end. Two guide RNAs, recognizing the IPO7 gene in exon 1 and exon 4, respectively, were used. The PAM sites are underlined and shown in green. The cleavage sites recognized by the nuclease of Cas9 are three nucleotides upstream of the PAM sites are shown in red arrowheads.

**Figure 2:**
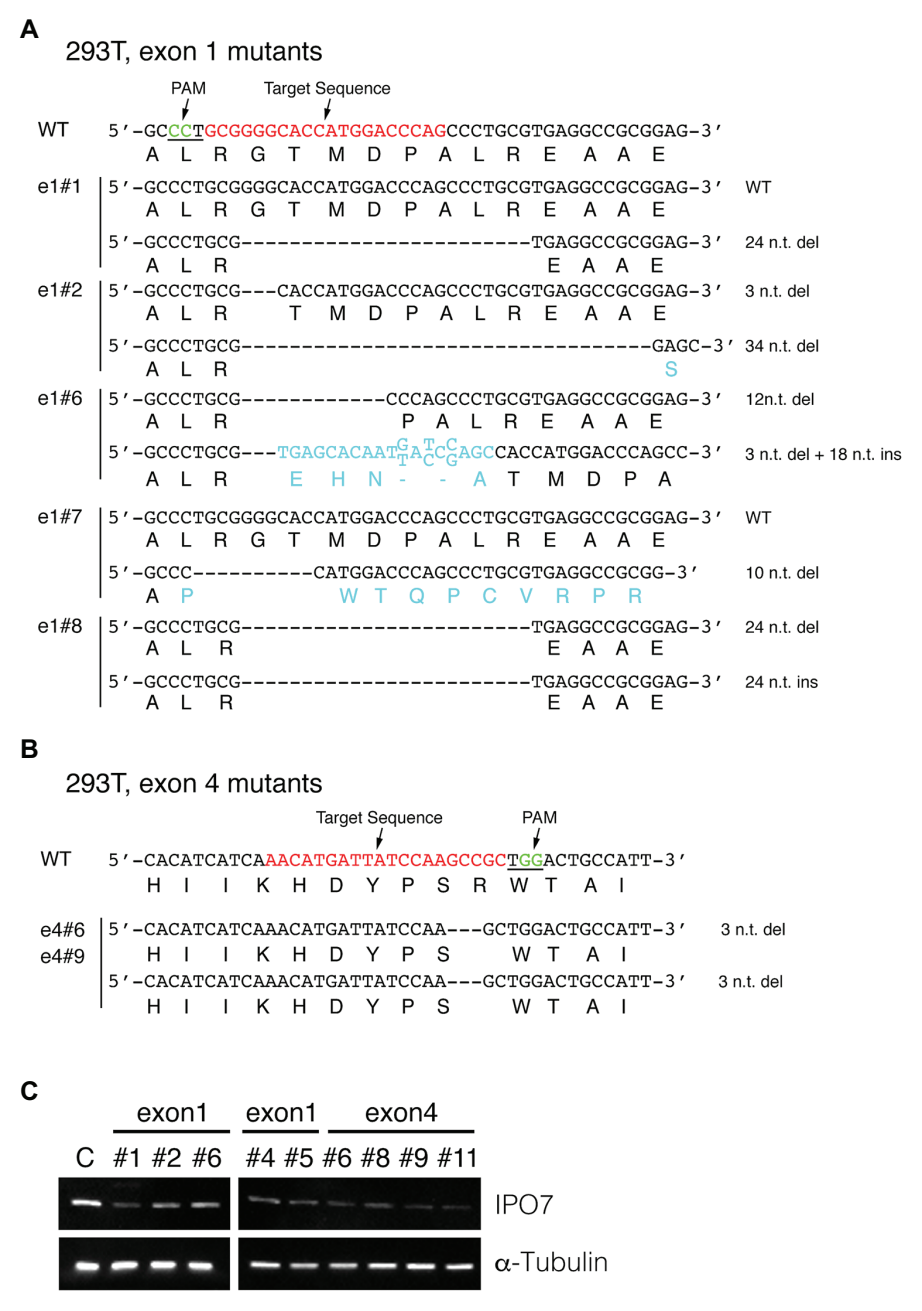
CRISPR-engineered IPO7-mutants in 293T cells. 293T cells were transfected with guide RNAs that targeted exon1 **(A)**, or exon4 **(B)** along with Cas9, and selected with Hygromycin for 3 weeks. The wild-type sequence is shown; the target sequence is shown in red and PAM underlined and shown in green. The sequence of each clone was read through sequencing and the mutated genomic sequence and amino acids are shown in blue. **(C)** The IPO7 expression of certain IPO7 mutants decreased as detected *via* Western blotting. The expression of α-Tubulin was measured as an internal control. The mean intensity of IPO7 in these clones was 0.31 relative to the control with a S.E. +/−0.14.

To elucidate any selective advantages EBV is afforded by inhibiting IPO7 in infected cells, we used CRISPR-Cas9-mediated genome editing to target the IPO7 gene in human cell lines with the goal of eventually expressing IPO7 conditionally. We used this approach also to avoid the off-targeting inherent to assays with siRNAs. While siRNAs recognize a desired target with perfect complementarity, they act through the miRNA pathway so that the “seed sequences” they necessarily encode direct them to other mRNAs, their “off-targets.” Such “off-targets” can be more robustly recognized that the desired target (Riba et al., 2017; Smith et al., 2017). Cas9 and guide RNAs targeting nucleotides 172~191 and 25,342~25,361 n.t., respectively, of IPO7 (Figure 1) were expressed with a stably replicating plasmid vector in 293T cells, which are EBV-negative, and two EBV-positive cell lines, 721 and SNU-719. Multiple clones were selected, and Sanger sequencing was performed to determine the editing of the targeted sites. Among 12 293T clones, five clones had no detected genomic changes in either allele; two of the remaining clones had a single mutated allele. These mutations were in-frame (e1#1), or frame-shifted with ten nucleotides deleted (e1#7). The remaining five clones had mutations in both alleles. One clone had an in-frame deletion of three nucleotides and an allele with a frame-shift mutation consisting of a deletion of 34 nucleotides (e1#2). The other four clones had each allele with in-frame mutations with deletions or insertions (clones e1#6, e1#8, e4#6, and e4#9; Figure 2; Table 1). We assayed the levels of expression of IPO7 in these clones. All of these clones expressed detectable but variable levels of IPO7 (Figure 2C) as did an additional four clones, e1#4, e1#5, e4#8, e4#11, in which the sequences of mutated alleles could not be determined. Thus in nine clones of EBV-negative, 293T cells, in which one or both alleles of IPO7 were successfully mutagenized, all expressed the protein detectably. This CRISPR-based method has been used successfully with analogous results to identify essential genes in other organisms (You et al., 2020). We conclude therefore that IPO7 is essential for the growth and or survival of 293T-cells in culture.

**Table 1:** CRISPR-engineered mutations in IPO-7 and allele classifications.

Cell type	WT	WT/Inframe mut	WT/Frameshift mut	Inframe mut/Frameshift mut	Inframe mut/Inframe mut	Total
293T	5	1	1	1	4	12
721	2	2	0	12	8	24
SNU-719	4	2	1	10	9	26
Total	11	5	2	23	21	62

**Figure 3:**
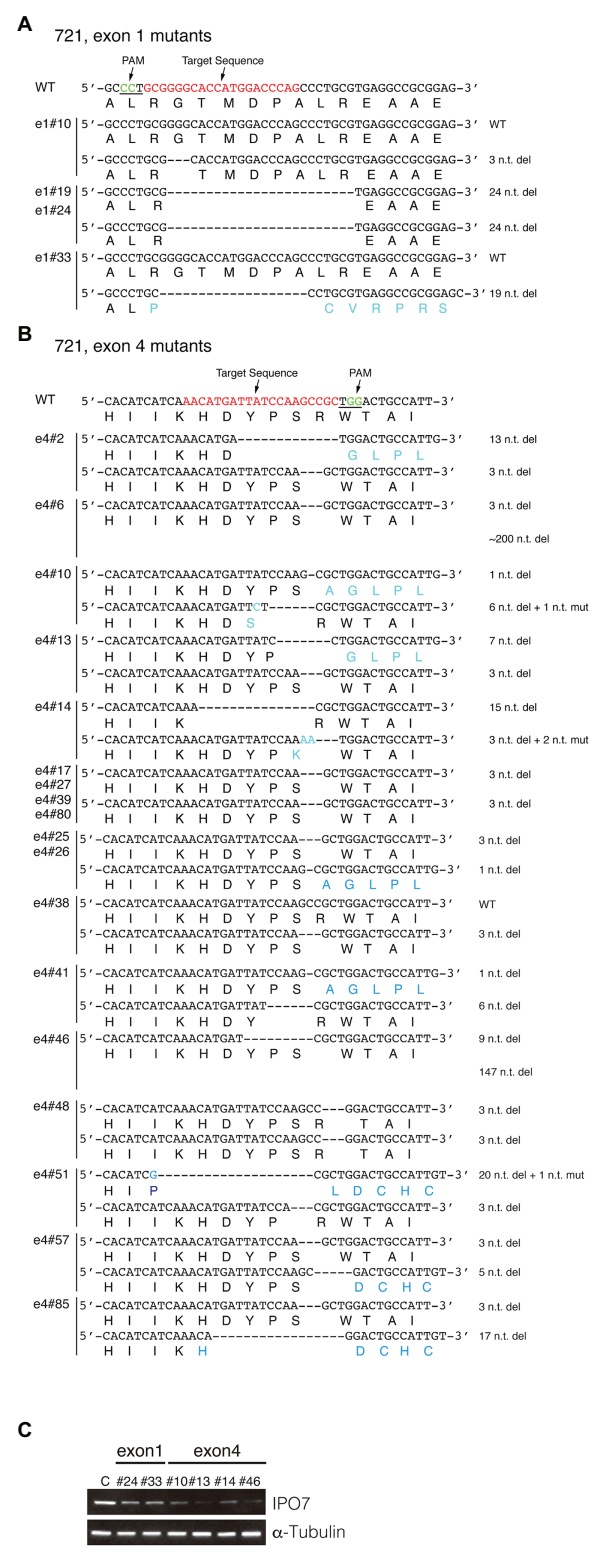
CRISPR-engineered IPO7 mutants in 721 cells. 721 cells were transfected with guide RNAs that targeted exon1 **(A)**, or exon4 **(B)** along with Cas9, as described in Figure 2. The wild-type sequence is shown; the target sequence is shown in red; and PAM is underlined and shown in green. The sequence of each clone was determined through sequencing and the mutated genomic sequence and its encoded amino acids are shown in blue. **(C)** The IPO7 expression of certain IPO7 mutants was decreased as detected *via* Western blotting. The expression of α-Tubulin was also determined as an internal control. The mean intensity of IPO7 in these clones was 0.22 relative to the control with a S.E. +/−0.07.

The same approach was used to mutagenize IPO7 in two EBV-positive cells, 721, a B-cell line and SNU-719, a gastric carcinoma cell line. Among 24 clones of 721 cells amenable to sequencing, two lacked genomic changes in each allele; two clones contained one wild-type allele and one allele with an in-frame mutation; and 12 clones contained one allele with an in-frame mutation and one allele with a frame-shift mutation; and eight clones had both alleles with in-frame mutations (Figure 3; Table 1). All of these clones expressed IPO7 detectably although most expressed it at lower levels than did the parental cells (Figure 3C). For example, even in clone e4#46, which contains one allele with a deletion of 147 nucleotides, IPO7 was detected faintly. Among the 22 mutant derivatives of 721 cells all contained at least one allele of IPO7 that could be translated into an expressed form of the protein. IPO7 is therefore also essential in EBV-transformed B-cells. In order to determine if these decreased levels of IPO7 affected the growth of 721 cells, the efficiency with which clones e1#24, e1#33, e4#14, and e4#46 survived cloning was tested. No differences between the parental 721 cells and their mutant progeny could be detected in this assay (data not shown) indicating that the activation of p53 leading to cell death observed in some cells with decreased levels of IPO7 (Golomb et al., 2012) does not occur in these EBV-transformed lymphoblasts.

**Figure 4:**
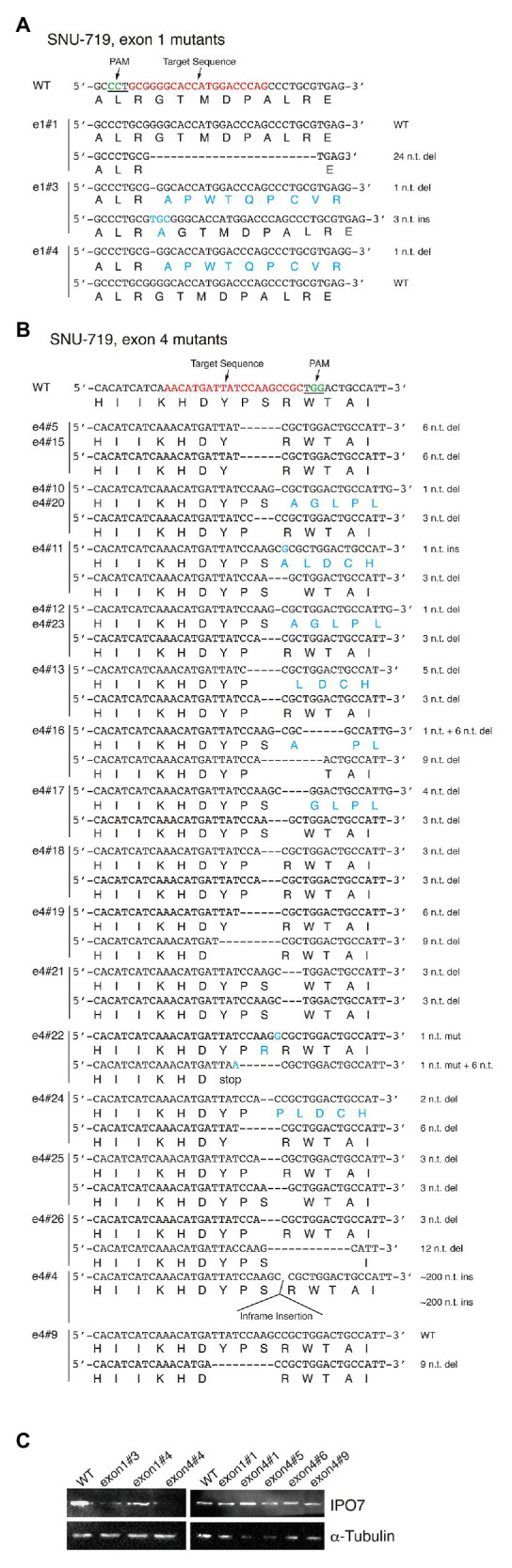
CRISPR-engineered IPO7 mutants in SNU-719 cells. SNU-719 cells were transfected with guide RNAs that targeted exon1 **(A)**, or exon4 **(B)** along with Cas9, as described in Figure 2. The wild-type sequence is shown; the target sequence is shown in red; and PAM is underlined and shown in green. The sequence of each clone was determined through sequencing and the mutated genomic sequence with its encoded amino acids are shown in blue. **(C)** The IPO7 expression of certain IPO7 mutants varied and was detected *via* Western blotting. The expression of α-Tubulin was also determined as an internal control.

Among 26 SNU-719 clones amenable to their alleles of IPO7 being sequenced, four clones had no genomic changes in either allele; two contained one wild-type allele and one allele with an in-frame mutation; one clone had one wild-type allele and one with a frame-shift mutation; 10 clones contained one allele with an in-frame mutation and one with a frame-shift mutation; and nine clones had two in-frame mutated alleles (Figure 4; Table 1). The IPO7 was examined by Western blotting, and some of these clones expressed IPO7 at lower levels than did cells with two wild-type alleles (Figure 4C). For two clones of SNU719 cells, clone e4#1 and e4#6, in which the sequences of mutated IPO7 could not be determined, IPO7 was still detected in a Western blot (Figure 4C). In the 28 characterized mutant derivatives of SNU-719 cells, all expressed IPO7 detectably indicating that some level of expressed IPO7 protein is essential for the growth and or survival of these cells.

One additional approach to testing if IPO7 is an essential gene in these cell lines is to determine the likelihood for the observed distribution of mutations in the 44 clones having mutations in both alleles (Table 1) arising by chance. In a simple, conservative model, in-frame mutations arise in one out of three contiguous deletions and out-of-frame deletions arise in two out of three of them. We used a permutation test to ask, given 88 alleles, of which 65 are in-frame and 23 are frame-shifted, if we randomly compute pairs over and over again, what is the fraction of the times in which frameshifts occur in both alleles? While we observed no such double frameshift, the permutation test predicted 1.9% would be double frameshifts yielding a value of *p* = 0.019. Again, we conclude that IPO7 is an essential gene in these three cell lines.

### Increasing Expression of IPO7 Inhibited the Cloning Efficiency of EBV-Positive Cells

**Figure 5:**
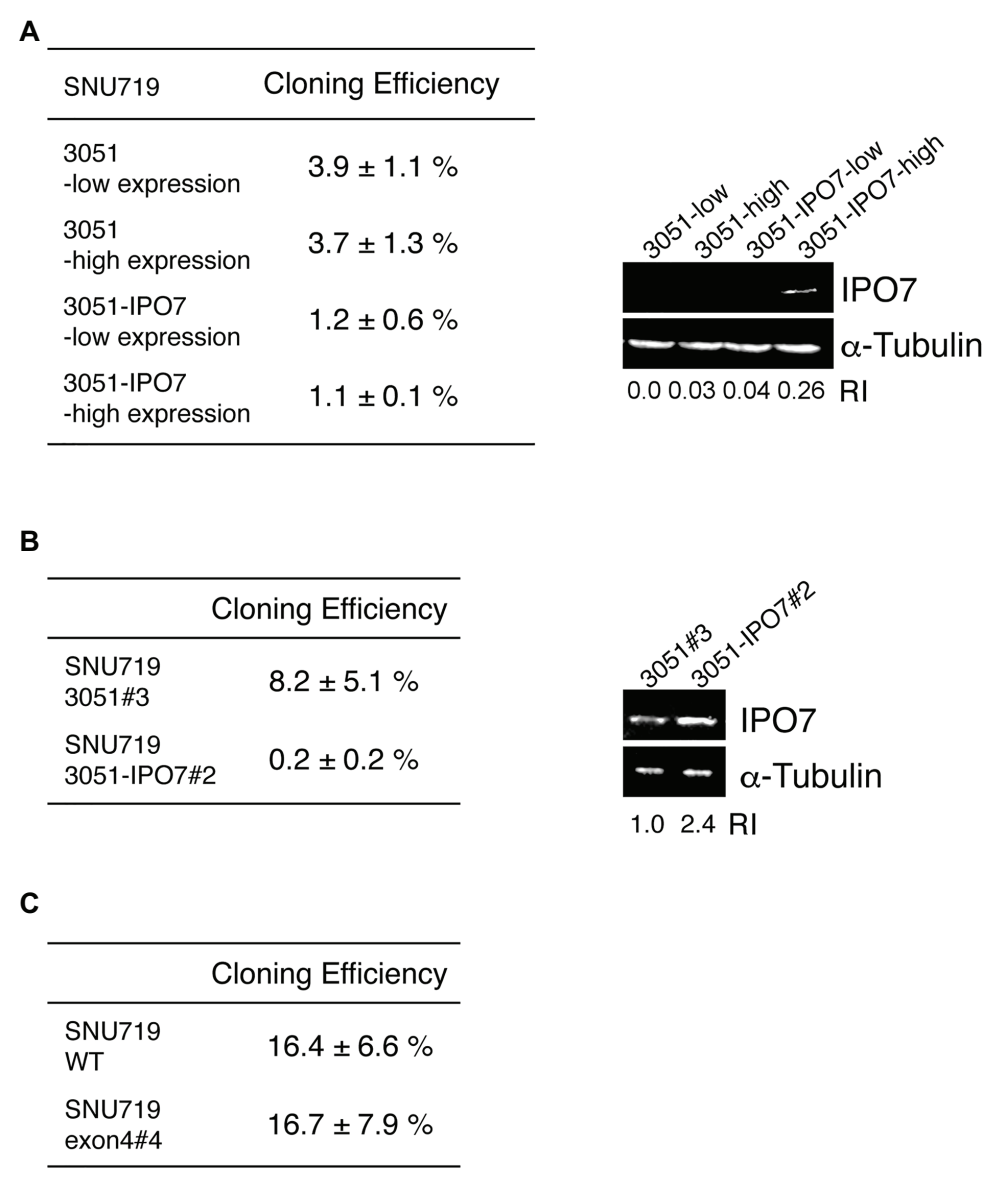
The effect of IPO7 expression levels on the cloning efficiency of SNU-719 cells. **(A)** SNU-719 cells were infected with a retrovirus carrying IPO7 (3051-IPO7) or an empty vector (3051). The infected cells were sorted by Flow cytometry as a function of the level of GFP expression. 30, 100, 300, or 1000 sorted cells were seeded on 10 cm plates and the cloning efficiencies measured after 1 month. The levels of IPO7 expression were detected *via* Western blotting analysis using a monoclonal IPO7 antibody. **(B)** A single clone of SNU-719 cells infected with the retrovirus carrying IPO7 (3051-IPO7, clone #2) and one infected with an empty vector (3051, clone#3) were used for cloning assays as described in **(A)**. The relative intensity of IPO7 expression were measured by ImageJ and normalized by α-tubulin expression. RI: relative intensity. **(C)** A single clone of SNU-719 cells (WT) or one carrying a CRISPR IPO7 mutant (exon4#4) were used for a cloning assay as described in **(A)**. All the cloning efficiencies were measured in more than three independent experiments. Errors are ±SE as an estimate of variation of mean of separate experiments.

Having found that IPO7 is essential for 293T cells and for EBV-positive cells, we asked if its increased expression was beneficial to one of the EBV-positive cells, SNU-719 cells, which are adherent and readily manipulated in culture. SNU-719 cells were infected with a retrovirus that expresses a bicistronic IPO7-IRES-GFP cassette, the infected cells were collected by sorting those with different levels of GFP, and further analyzed by cloning. SNU-719 cells with varied levels of IPO7 expressed from this vector had decreased efficiencies of cloning relative to SNU-719 cells infected with the empty vector (Figure 5A). We confirmed this finding with bulk populations of retrovirus-infected cells by picking clones of infected cells and testing their cloning efficiencies. A clone infected with the retrovirus that expressed IPO7 at a 3-fold higher level than a clone infected with the empty virus had 40-fold lower cloning efficiency (Figure 5B). We also tested if a decreased expression of IPO7 affected the survival of these cells. The cloning efficiency of one IPO7-CRISPR mutant, SNU719 e4#4, was determined and compared with that of a wild-type clone. SNU719 e4#4 had a lower expression of IPO7 (Figure 4C) and gave the same cloning efficiency as that of wild-type cells (Figure 5C).

Increasing the expression of IPO7 modestly in a bulk population of SNU-719 cells diminished their cloning; increasing it several fold inhibited cloning of these cells dramatically (Figures 5A,B). These findings are complemented by the findings with the EBV-positive 721 cells in which four clones with decreased levels of IPO7 were found to clone as efficiently as did the parental cell. While IPO7 is essential for EBV-positive cells, lower than wild-type levels of expression are tolerated, but higher than wild-type levels are deleterious to their growth and/or survival.

### IPO7 Causes the Accumulation of EGR1 in the Nucleus

**Figure 6:**
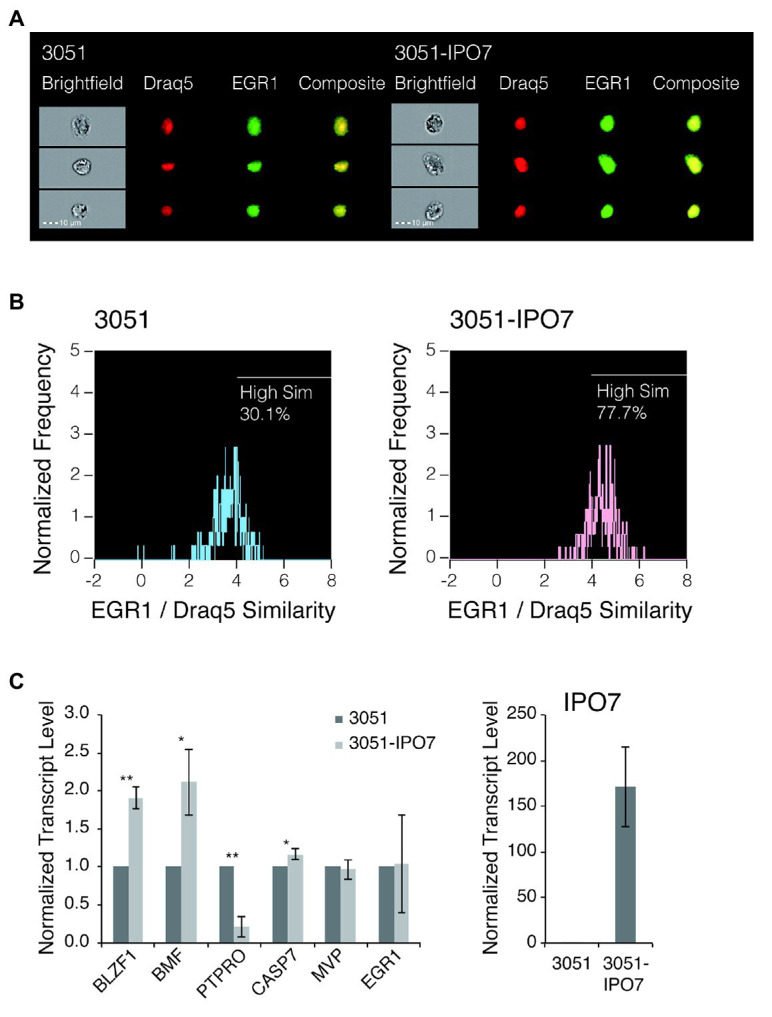
The nuclear accumulation of EGR1 in SNU-719 cells with high level IPO7 expression. **(A)** SNU-719 cells transfected with 3051 or 3051-IPO7 were fixed and stained for EGR1 and with Draq5 (Draq5 is a nuclear stain that tags its localization). The cell suspensions were run on ImageStream. Brightfield (white), EGR1 (green), Draq5 (red), and EGR1/Draq5 composite images for three representative cells (of 1,000 imaged) are shown for each transfection sample. **(B)** The EGR1/Draq5 similarity score from 1,000 images of each transfection sample were analyzed by ImageStream and depicted in the histogram. The percentage of cells that fall within a High Similarity region are drawn. **(C)** SNU-719 cells transfected with 3051 or 3051-IPO7 were sorted based on their EGFP signal intensities. Total RNAs were isolated and reverse transcribed. The transcript levels of the EGR1-regulated genes, including BLZF1, BMF, PTPRO, CASP7, and MVP were measured with quantitative PCR. The transcript level of EGR1 was also measured. The transcript level of IPO7 was measured to confirm the overexpression of IPO7. The error bars indicated the SD. *p* values were calculated by Student’s *t* test. ^*^p < 0.05; ^**^p < 0.001.

Previous studies found that IPO7 is critical for the translocation of EGR1 into the nucleus (Fassati et al., 2003; Ao et al., 2007). The expression of EGR1 increases when EBV’s lytic phase is induced by treating B-cells with anti-immunoglobulin (Ye et al., 2010) and its transcription is increased by EBV’s early gene, BZLF1, which also fosters the induction of EBV’s lytic phase (Chang et al., 2006; Heather et al., 2009). It is therefore reasonable to hypothesize that the regulation of IPO7 in turn regulates the level of EGR1, which is one determinant of the EBV’s entry into its lytic phase. We tested this hypothesis by measuring the localization of EGR1 to the nucleus in cells expressing different levels of IPO7 and the effects of this nuclear localization on genes regulated by EGR1. To do so, SNU-719 cells were transfected with an empty retroviral vector, 3051, or 3051 expressing IPO7 and the degree of nuclear translocation of EGR1 was measured by ImageStream. This approach allowed us to assay a thousand immunochemically stained cells and a thousand control cells. Localization of the immunochemically stained EGR1 to the nucleus is reflected by the “similarity score” (George et al., 2006). A higher positive value of the similarity score means that the florescent signal of EGR1 and the nuclear image are more alike, indicating that more EGR1 is localized to the nucleus. The measurements showed that a greater portion of EGR1 distributed to the nucleus and exhibited a similarity score greater than 2 in SNU-719 cells that expressed 3051-IPO7 relative to these cells with the empty vector (Figures 6A,B). When cells overexpressing IPO7 were compared to those expressing the empty vector, 2.6-fold more EGR1 was localized to the nuclei and these cells exhibited a similarity score > 4. The measurement of EGR1’s movement to the nucleus with high levels of IPO7 is robust as reflected by similar measurements of I*κ*B*α* with or without its nuclear localization sequence localizing to the nucleus. This assay found less than a 2-fold difference in the similarity score for these two conditions (Lisiero et al., 2020). Increasing expression of IPO7 thus contributes to increasing nuclear localization of EGR1.

To determine if increased nuclear localization of EGR1 leads to changes in the expression of genes it regulates in EBV-positive cells, we considered candidates identified previously by ChIP-on-ChIP assays performed in prostate tumor cells (Arora et al., 2008). EGR1 can enhance the transcription of a gene’s promoter directly or inhibit a gene’s expression indirectly, for example, by enhancing expression of specific miRNAs (Xu et al., 2015). We screened the candidates for ones differentially expressed in B-cells transformed with a wild-type strain of EBV vs. B-cells transformed by a strain of EBV deleted for its miRNAs (Tagawa et al., 2016) to focus on candidates potentially affected by differing levels of expression of IPO7. We then assayed some of these candidates in SNU-719 cells transfected with the empty vector, 3051, or the vector expressing IPO7, 3051-IPO7. Three of these genes, Protein Tyrosine Phosphatase Receptor Type O (PTPRO), a member of the transmembrane receptor family of protein tyrosine phosphatases (Motiwala et al., 2003, 2004; You et al., 2012), basic leucine zipper nuclear factor 1(BLZF1; Tong et al., 2000), and Bcl-2 modifying factor(BMF; Frenzel et al., 2010; Baumgartner et al., 2013) were altered in their expression with PTPRO decreasing and both BLZF1 and BMF increasing by a factor of 2 (Figure 6C). The levels of two other candidates, Caspase 7 and Major Vault Protein, were not altered between these cells (Figure 6C). Although it is unclear whether the changes in expression of BLZF1, BMF, and PTPRO contribute to the deleterious effects in EBV-positive cells when levels of IPO7 are increased, increasing the level of IPO7 increases the level of EGR1 transported into nucleus without increasing the accumulation of its RNA (Figure 6C). The resulting increases in the nuclear localization of EGR1 correlate with significant changes in the levels of some genes that are candidates for being regulated by EGR1 in EBV-positive cells indicating that not only is more EGR1 moving to the nucleus but also appears to be regulating transcription there, too.

We independently confirmed this coupled analysis of ImageStream and mRNA measurements of EGR1 transcriptional targets. We assessed the levels of the RNAs encoding the relevant genes in cells infected with wild-type EBV or with a mutant lacking its miRNA genes (Tagawa et al., 2016). In the cells infected with wild-type EBV and its miRNAs, IPO7 was detected in the RISC complex and was reduced significantly to 0.82 of wild-type levels. In the presence of EBV’s miRNAs, EGR1 mRNA levels remained the same but the levels of its two transcriptional targets, BMF and BLZF1, were decreased significantly to 0.75 and 0.81 of the levels measured in the absence of the miRNAs, respectively. These two mRNAs were not detected in the RISC complex consistent with their decreased levels resulting from less EGR1 being transported into the nucleus by IPO7.

## Discussion

Several large-scale screens have found the transcripts of IPO7 to be frequent targets for EBV’s miRNAs including BART3 and BART16 (Riley et al., 2012; Skalsky et al., 2012; Vereide et al., 2014). Multiple studies have also found that EBV’s targeting specific cellular RNAs with its miRNAs provides EBV selective advantages by, for example, avoiding apoptosis or evading a host’s immune responses (Marquitz et al., 2011, 2012, 2014; Riley et al., 2012; Vereide et al., 2014; Cai et al., 2015; Kanda et al., 2015; Kang et al., 2015; Lin et al., 2015; Shinozaki-Ushiku et al., 2015; Albanese et al., 2016; Tagawa et al., 2016; Zhang et al., 2018; Murer et al., 2019; Wang et al., 2019). We have asked if EBV’s targeting IPO7 provides EBV-infected cells a selective advantage, and if so, what that advantage might be. Because IPO7 contributes to the nuclear import of many proteins (Pumroy and Cingolani, 2015), it is unlikely that any possible advantage gained by targeting IPO7 can be ascribed to only one of IPO7’s cargoes. In addition, several different importins often transport the same protein into the nucleus (Pumroy and Cingolani, 2015) possibly confounding our identifying any advantage EBV derives from targeting IPO7. We first attempted to delete the IPO7 gene from the EBV-negative 293T cell line, from an EBV-positive B-cell line, 721, and from an EBV-positive gastric carcinoma cell line, SNU-719 using CRISPR/Cas9. We found that among 44 clones with mutations in both alleles of IPO7 in these three cell types (Table 1) all expressed IPO7 detectably indicating that IPO7 is an essential gene in these cells. This conclusion is supported by a permutation test in which we randomly computed pairs of alleles over and over again and asked what is the fraction of the times in which frameshifts occur in both alleles based on our experimental finding of 23 of 88 alleles with frameshifts? While we observed no such double frameshift, the permutation test predicted 1.9% would be double frameshifts yielding a value of *p* = 0.019. It is surprising, perhaps, that EBV uses its miRNAs to target the mRNAs of an essential gene. However, EBV’s targeting IPO7 is substantiated by two sets of functional analyses: First, evicting EBV from Burkitt Lymphoma cells leads to their levels of the IPO7 protein being increased, while re-expression of the viral miRNAs in these evicted cells leads to IPO7 being decreased to parental levels (Vereide et al., 2014). Second, infecting primary B-cells with EBV or a derivative from which its miRNA genes were deleted shows that the absence of EBV’s miRNAs in newly infected B-cells leads to a significant increase in the expression of IPO7 mRNA (Tagawa et al., 2016).

Inhibiting IPO7 reduces the nuclear import of some ribosomal proteins, leads to the activation of p53 and decreases the survival of some cell types (Golomb et al., 2012). We measured the survival of EBV-positive B-cells and EBV-positive gastric carcinoma cells in which the levels of IPO7 were demonstrably reduced following mutagenesis by treatment with CRISPR/Cas9 (Figures 3C, 4C) and found no effect on their survival. Given that in these experiments, the levels of IPO7 were often decreased relative to those in the parental EBV-positive cells, we can conclude that the levels of IPO7 achieved by EBV’s miRNAs in these lymphoid and epithelial cells do not activate p53 to promote cell death. cMyc stimulates the expression of IPO7 (Golomb et al., 2012) and EBV genes can regulate the expression of cMyc. For example, the conditional expression of the LMP-1 gene of EBV in B-cells induces the expression of cMyc (Dirmeier et al., 2005). Perhaps, EBV-transformed lymphoid and epithelial cell lines use multiple paths to ensure that the expression of IPO7 is consistent with their survival. Given that EBV-transformed cells can accommodate decreased levels of IPO7, we asked if they could also accommodate increases in its expression. A vector expressing IPO7 or an empty vector was introduced into SNU-719 cells and populations or clones of these cells were tested for their survival by measuring the efficiencies of their cloning. The added IPO7 functioned in these cells. The increased levels of IPO7 increased the nuclear import of its cargo as measured by the increased nuclear localization of EGR1 and alterations in the expression of some genes known to be regulated by EGR1 (Figure 6). These increases in IPO7 inhibited the cloning efficiencies of both the bulk populations of cells and a clone of them (Figure 5) thus limiting proliferation. We conclude that EBV targets IPO7 with its miRNAs to cap the expression of IPO7 at a level optimal for the growth of EBV-transformed cells.

## Data Availability Statement

The datasets presented in this study can be found in online repositories. The names of the repository/repositories and accession number(s) can be found in the article/supplementary material.

## Author Contributions

Y-CY and BS designed experiments, interpreted data, and wrote the manuscript. Y-CY performed experiments. All authors contributed to the article and approved the submitted version.

### Conflict of Interest

The authors declare that the research was conducted in the absence of any commercial or financial relationships that could be construed as a potential conflict of interest.
